# Proximate composition of *Vitex doniana* and* Saba comorensis* fruits

**DOI:** 10.1038/s41598-023-46874-7

**Published:** 2023-11-23

**Authors:** Dominic Charles, Clarence Mgina

**Affiliations:** Chemistry Department, University of Dar es Salaam, Dar es Salaam, Tanzania

**Keywords:** Biochemistry, Health care

## Abstract

The wild fruits of *Vitex doniana* and *Saba comorensis* were randomly collected from Pwani and Tanga regions. Laboratory analysis was done using the methods described by the Association of Official Analytical Chemists AOAC (1995 and 2000). The amount of protein was 7.13 ± 0.04% and 21.73 ± 0.02% in *V. doniana* fruits while the fat contents were 2.4 ± 0.00% and 1.9 ± 0.10% in *V. doniana* fruits. The amount of fats in *S. comorensis* fruits ranged from 0.00 to 0.01% for the fruit samples from both Pwani and Tanga regions, however the differences was not statistically significant (*P* > 0.05). The amounts of carbohydrates in *V. doniana* 23.98 ± 0.20% and in *S. comorensis* fruit samples (23.81 ± 0.38%) from Pwani Region were not statistically difference. The differences can be attributed to environmental and soil factors. *S. comorensis* fruit samples from Tanga had ash 4.20 ± 0.01% and moisture content 70.97 ± 0.04%. These values were higher than those observed for *S. comorensis* fruit samples from Pwani. The amount of ash is indicative of potential elements like sodium and potassium which are beneficial in human health for the development of bones. These fruits have significant amounts of carbohydrate and protein and hence healthy for consumption as part of human diet.

## Introduction

Wild fruits have potential nutritive components that are very beneficial to human health^1^, they can be defined as whole or their any part(roots, leaves or fruits) that are edible^2^. Many of rural people consume wild fruits due to easy access while urban people eat cultivated exotic fruits that are easy available from markets^3^. In addition rural communities depend on wild fruits to meet their food needs during food crisis^3^. Majority of rural inhabitants depend on the natural resources where there is high availability of diverse trees that bear edible wild fruits. Pawlos et al.^4^ revealed that wild foods are seasonal although, they play a great role in human diet supplying the body with nutrients, vitamins, proteins and carbohydrates. Most of these wild fruits have high nutritive values than exotic fruits commonly sold in market^5^.

According to^6^, twenty million children suffer from severe acute malnutrition which results into one million deaths each year. One of the major causes of malnutrition is the lack of essential nutrients in diet. Wild fruits have proven to compose nutrients such as carbohydrates, proteins, minerals, vitamins and fats for curbing malnutrition^1,7^. Moreover, wild fruits have been found to have higher nutritive values than some exotic ones and are mostly used by communities^7^. There has been a growing interest in determining the nutritional composition of different wild fruits that potentially may have high nutritional values^8–10^.

This study therefore intended to evaluate the nutritive values of these wild fruits that can contribute to widen nutritional sources. The soft fleshy parts (mesocarp and exocarp) of *Vitex doniana* are the most preferred while the seeds are discarded. The mesocarp of *Saba comorensis* commonly known as rubber vine, is cut open and eaten as a snack together with its seed. The ripe fruits are collected between the end of the rainy season (January) and the beginning of the dry season (march) in Tanzania^3^. *V. doniana* and *S. comorensis* fruits are in danger due to anthropogenic activities, therefore there is a need to be evaluated and documented by shown their advantages to human health and calling the preservation of these wild fruits in coastal regions of Tanzania as the have started to be threatened and soon without any action they will be extinct.

Most of the edible wild fruits are collected for consumption as food, snack and juices. In this study determined the nutritional composition including carbohydrate, protein and fats of *V. doniana* and *S. comorensis* fruits from coastal forests in Tanzania. To the best of my knowledge little is still know for the nutritional profile of *V. doniana* and *S. comorensis* fruits from coastal forests of Tanzania.

## Material and methods

### Sampling of wild fruits

Sample collection was permitted by the institution of Dar es salaam following the guidelines to comply with conversion on trade in endangered species of wild fauna and flora. A total of 240 Fruit samples of *S. comorensis* were collected randomly from four areas that included Kibiti (60 fruits), Ngomboloni (60 fruits), Mkuranga (60 fruits) and Nyakikai (60 fruits) in the coastal forests. From Tanga region total of 180 fruits samples were taken randomly from different local markets that obtained them from nearby coastal forests. *V. doniana* fruits were also collected from four areas that included Kibiti, Ngomboloni, Mkuranga and Nyakikai in the coastal forests. From Tanga region the fruits were taken randomly from different local markets that obtained them from nearby coastal forests. The mature and healthy ripe fruits of *S. comorensis* and *V. doniana* were collected in March and April 2019. The fruits were identified by a botanist (Mr. Charles) from the herbarium of the Botany Department at University of Dar es Salaam and voucher specimen has been deposited at University of Dar es salaam (Botany Department).

### Sample preparation

The morphological characterization of fruit samples including weight and pulp weight of ripe fruits were carried out in the laboratory. An electrical analytical balance (Shimadzu ATY224; Japan) was used to measure the weight (g) of each fresh fruit and the mean weight was calculated and recorded. The weighed fruits were divided into two portions by cutting them into half using a stainless-steel knife. The pulps and seeds were removed by using a metal spoon. The pulp together with seeds were put in a dish and blended manually to separate the pulp and seeds. The seeds with residual pulp were dried in an oven at 60 oC for 5 h to completely separate seeds from residue pulp. The seeds were separated by peeling off the pulp residue and the edible parts were homogenized using mortar and pestle. The weight (g) of the pulp was measured by using an analytical balance and the mean weight was calculated.

**Figure 1:**
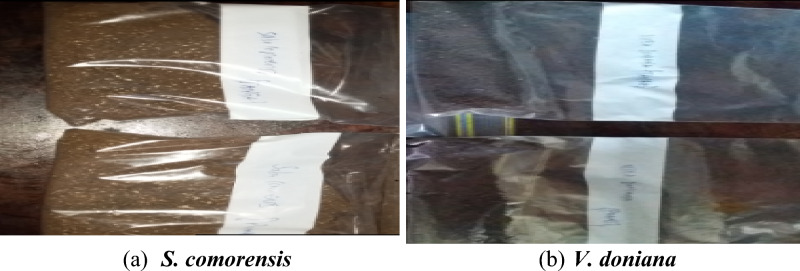
The powder of wild fruits.

The moisture and ash contents of the fresh fruits pulp were determined. The edible part of the fruits (mesocarp) was dried in shades for two weeks then taken to the oven overnight 65 °C. After drying the sample was ground to a powder (Fig. 1) and then analyzed for its crude fat and protein.

### Physical–chemical analysis of wild fruits

#### Determination of moisture content

The moisture content was determined using methods described by^14,15^. A clean dish was dried in an oven at 105 °C for 30 min and then cooled in a desiccator. The empty dish was weighed and 40 g of the sample was weighed and put into a dish (*w*1 g). The dish with a sample were then heated at 105 °C for 30 min and then cooled in desiccator. The sample was weighed (*w*2g) and the moisture content was calculated by using the following Eq. 1.

1 $$\% {\text{ moisture}}\,{\text{content}} = \frac{{{\text{Loss}}\,{\text{in}}\,{\text{weight}}\left( {{\text{w}}1 - {\text{w}}2} \right)}}{{{\text{Initial}}\,{\text{weight}}\,{\text{of}}\,{\text{the}}\,{\text{sample}}\left( {{\text{w}}1 - {\text{w}}} \right)}} \times 100$$

where by, Weight of the empty dish = (*w* g); Weight of dish + samples before oven drying = (*w*1g); Weight of the dish + sample after drying in oven = (*w*2g).

#### Determination of ash content

The method used was described by^15^. The crucible was placed on the furnace at 500 °C for 24 h to ensure that impurities on the surface of crucible are completely removed. The crucible was then cooled in the desiccator for about 30 min and then weighed. Five grams (5 g) of the sample was placed in the crucible (w3) and then heated in a furnace at 500 °C for 24 h. The sample was then cooled in the desiccator until it turns grey. The crucible with the sample and lid were weighed and calculated for ash content using Eq. 2.

2 $${\text{The}}\,{\text{percentage}}\,{\text{of}}\,{\text{ash}}\,{\text{content}} = \frac{{{\text{Weight}}\,{\text{of}}\,{\text{ash}}\left( {{\text{w}}3 - {\text{w}}} \right)}}{{{\text{Weight}}\,{\text{of}}\,{\text{sample }}\left( {{\text{w}}1 - {\text{w}}} \right)}} \times 100$$

where by, Weight of the dish + weight of dried sample = (*w*1 g); Weight of dish + weight of ash = (*w*3 g).

#### Determination of crude fat content

Crude fat content was determined using method described by^15^. Fifty gram (50 g) of the sample was weighed (W_1_ g) and transferred into the extraction thimble and placed in its siphon height. The weighed extraction flask was connected to the extractor carrying the thimble and 200 mL of petroleum ether was heated for 4 h. The solvent was allowed to vaporize at 60 °C and then condensed and allowed to fall drop-wise into the thimble to extract fats present in a sample. The flask with the extract was removed and the extracts were concentrated using vacuum rotary evaporator, cooled in desiccators and weighed (W_2_ g). The percentage yield of ether extract was calculated based on Eq. 3.

3 $${\text{The}}\,{\text{percentage}}\,{\text{of}}\,{\text{the}}\,{\text{sample}} = \frac{{{\text{w}}2 - {\text{w}}1}}{{\text{w}}} \times 100\%$$

where by, Weight of sample to be used*(w);* Weight of the dry flask *(w*1*);* Weight of the flask + fat after evaporation and cooled *(w*2*).*

#### Determination of crude protein

The crude protein was determined by conversion of organic nitrogen to ammonia based on Kjeldah method described by^16^. 0.100 g of dried and finely ground sample was weighted into a 50 mL Kjeldahl flask. Two grams of potassium sulphate and copper sulphate mixture 1:1 was added into a flask. This was followed by adding 3 mL of concentrated Sulphuric acid slowly down the neck while rotating the flask and then heating gently until frothing subsided. After the digest became colorless, it was heated for 30 min to completion and allowed to cool. The digest was then diluted to 50 mL with distilled water and analyzed for nitrogen as ammonia nitrogen through spectrometric method with color reactions at 660 nm (indophenol – blue method).

Nitrogen standard (1 mL = 0.1 mg NH_4_^+^–N) and working standard (1 mL = 0.001 mg NH_4_^+^–N) were prepared from stock solutions. The combined reagent was prepared by dissolving 35 g of sodium potassium tartrate, 17.5 g sodium salicylate and 0.5 g sodium nitroprusside in 400 mL water. Then 40 mL of 50% sodium hydroxide was added, mixed and stored at 2 °C. Sodium hypochlorite solution was prepared and then used to oxidize the ammonium–nitrogen. 10 mL of the working standard was pipetted into 50 mL volumetric flask to give a range from 0 to 0.001 mg NH_4_^+^–N. The blank was added to match the sample aliquots. 5 mL of a sample was pipetted into 50 mL volumetric flask, 40 mL combined reagent was added to both standard and the sample and also 4 mL sodium hypochlorite reagent was added and diluted to volume. Then the mixture was left in the water bath at 40 °C for 10 mn. The absorbance was measured at 660 nm using UV–VIS spectrophotometer (SPECRO-UK 6305).

A calibration curve was prepared from the standard values of nitrogen and used to obtain mg of NH^4^ + –N in the sample aliquot. The nitrogen (%) was calculated by using the following Eq. 4;

4 $${\text{Nitrogen}}\left( \% \right) = \frac{{{\text{c}}\left( {{\text{mg}}} \right) \times {\text{solution}}\,{\text{vol}}\left( {{\text{ml}}} \right)}}{{100 \times {\text{aliquot}}\left( {{\text{ml}}} \right) \times {\text{sample}}\,{\text{wt}}\left( {\text{g}} \right)}}$$

C = mg NH^4^ + –N obtained from the graph.

The crude protein (%) obtained by multiplying total nitrogen by conversion factor 6.25 (Eq. 5).

#### Determination of carbohydrate

Total carbohydrate was calculated by subtracting the sum of percentage of protein, fat, ash and moisture. Carbohydrate is divided into two groups’ crude fiber and nitrogen free extract (NFE). In this study only carbohydrate with crude fiber was determined, carbohydrate includes crude fiber was calculated by subtracting the sum of percentage of ash, fat, protein and moisture from hundred. The carbohydrate content was obtained by subtracting all values obtained from moisture, crude oil, crude protein, crude fiber and ash content from 100^17^.

5 $$100 - \left( {{\text{weight in grams }}\left[ {{\text{protein}} + {\text{crude oil}} + {\text{moisture}} + {\text{ash}}} \right]{\text{ in 1}}00\,{\text{g}}\,{\text{of sample}}} \right).$$

### Data analysis

Data was analyzed by using t-test from the statistical software known as Paleontological Statistics (PAST) version 2.17. The significant different was assessed at 5% critical value.

## Results

### The moisture content in the fruits of *V. doniana* and *S. comorensis*

The percentage moisture content in *S. Comorensis* fruits ranged between 65.48 and 65.80% for the fruit samples from Pwani and between 70.83 and 71.01% for the fruit samples from Tanga (Table 1). These differences were however statistically not significant (t = − 0.7003, df = 2, *P* > 0.05). The moisture contents in *V. doniana* fruits ranged from 64.51 to 64.73% for samples from Pwani and 64.51% to 64.73% for the fruit samples from Tanga. Similarly, these differences were not significant (t = − 1.1958, df = 2, *P* > 0.05) (Table 2).

**Table 1:** Physical–chemical properties of *S. comorensis* fruits.

Parameter	*S. comorensis* from Pwani	*S. comorensis* from Tanga	t-value	*P* value	Conclusion
Moisture content (g/100 g)	65.64 ± 0.16	70.97 ± 0.04	0.7003	*P* > 0.05	Not significant
Total ash (g/100 g)	5.35 ± 0.00	4.20 ± 0.01	1.7344	*P* > 0.05	Not significant
Crude protein (g/100 g)	5.20 ± 0.21	6.02 ± 0.17	5.1486	*P* < 0.05	Significant
Crude fat(g/100 g)	0.00 ± 0.01	0.00 ± 0.00	1.1094	*P* > 0.05	Not significant
Total carbohydrate (g/100 g)	23.81 ± 0.38	18.815 ± 0.22	0.7859	*P* > 0.05	Not significant

t (0.05) (2), 2 = 4.303.

**Table 2:** Physical–chemical Properties of *V. doniana* Fruits.

Parameter	*V. doniana* from Pwani	*V. doniana* from Tanga	t-value	*P* value	Conclusion
Moisture content (g/100 g)	64.67 ± 0.16	65.79 ± 0.08	1.1958	*P* > 0.05	Not significant
Total ash(g/100 g)	1.82 ± 0.00	1.86 ± 0.00	0.1574	*P* > 0.05	Not significant
Crude protein (g/100 g)	7.13 ± 0.04	21.73 ± 0.02	94.704	*P* < 0.05	Significant
Crude fat (g/100 g)	2.4 ± 0.00	1.90 ± 0.10	1.5443	*P* > 0.05	Not significant
Total carbohydrate (g/100 g)	23.98 ± 0.20	8.72 ± 0.40	0.1112	*P* > 0.05	Not significant

t (0.05) (2), 2 = 4.303.

### The amount of ash content in *V. doniana* and *S. comorensis*

The percent of ash content in *S. comorensis* fruit samples ranged from 3.19 to 4.21% for fruit samples from Tanga and 5.35% to 5.35% for samples from Pwani (Table 1). However, the difference between the two regions was not significant (t = 1.7344, df = 2, *P* > 0.05). Ash content in *V. doniana* fruit samples ranged between 1.82 ± 0.00 and 1.86 ± 0.00% for the fruit’s samples from Pwani and Tanga regions, and the difference between the two regions was not significant (t = 0.1574, df = 2, *P* > 0.05) (Table 2).

### The amount of crude protein content in *V. doniana* and *S. comorensis*

A range of 7.08% to 7.16% was observed for the amount of protein in *V. doniana* fruit samples from Pwani and 21.71% to 21.75% for the fruit samples from Tanga. The difference of crude protein contents in *V. doniana* fruits between the two regions based on two sample t-test was significant (t = 94.704, df = 2, *P* < 0.05). In this study *S. comorensis* fruits had an amount of crude protein in a range between 4.99 and 5.41% for the fruit samples from Pwani and between 5.85 and 6.19% for the fruit’s samples from Tanga. The differences in ash contents on *S. Comorensis* fruits from the two regions based on two sample t-test was also significant (t = 5.1486, df = 2, *P* > 0.05) (Table 1).

### The amount of crude fat content in *S. comorensis* and *V. doniana*

The amount of fats in *S. comorensis* fruits ranged from 0.00% to 0.01% for the fruit samples from both Pwani and Tanga. For *Vitex doniana* fruits the range was between 1.80% to 2.00% for the fruit samples from Tanga and 2.4 ± 0.00 for the fruit samples from Pwani. For both *S. comorensis* (t = -1.1094, df = 2) and *V. doniana* (t = 1.5443, df = 2) the differences between the Tanga and Pwani were not significant (*P* > 0.05).

### The total carbohydrate content in *S. comorensis* and *V. doniana*

The amount of carbohydrate in *S. comorensis* fruit samples ranged from 23.43 to 24.19% for the fruit samples from Pwani region and 18.60–19.04% for the fruit samples from Tanga region with no significant difference between samples from the two regions based on two sample t-test (t = -0.7859, df = 2, *P* > 0.05) Table 1. Carbohydrate contents in *V. doniana* fruit samples ranged between 23.68% and 24.18% for the fruit samples from Pwani region and between 8.32% and 9.12% for the fruit samples from Tanga region. Again, the difference of carbohydrate content between two regions was not significant (t = 0.1112, df = 2, *P* > 0.05) (Table 2).

## Discussion

### Variation of moisture content in the fruits of *V. doniana and S. comorensis*

The moisture content in *S. comorensis* fruits is similar to the moisture content in other wild fruits reported by^18^ in *Baccaurea ramiflora* (70.21%). Bamigboye et al.^17^ pointed out that the lowest moisture content signifies the highest dry matter content in fruits, therefore high moisture content in *S. comorensis* reveals lowest dry matter content.

However, moisture content in *Vitex doniana* was close to that in *Polyalthia suberosa* (64.76 ± 3.91%) reported by^19^. In this study values were higher compared to those in *Vitex doniana*, *Vitex kiniensis* and *Vitex fischerii* reported by^20^ that had the value of 39.42 ± 0.72%, 40.56 ± 0.77% and 37.74 ± 0.76%, respectively, this may be due to differences in climatic condition. The lowest moisture content in *V. doniana* fruits can favour long shelf life because the growth of microorganisms are not favored^21^. The percentage of moisture content in *V. doniana* and *S. comorensis* was lower compared to that in *Bridela tomentosa* and *Carissa spinarum*, 78.54 ± 1.02% and 73 ± 1.37% respectively as reported by^19^. Also some of the domesticated fruits such as *Mangifera indica* (82.1%) reported by^22^, *Passiflora edulis* (83.11%) reported by^12^ and *Citrus sinensis* (87.1%) reported by^13^ have higher moisture content than wild fruits.

Determining moisture content is important in food quality analysis because moisture affects preservation and resistance to deterioration^21^. The percentage of moisture content affects the physical and chemical properties such as color and taste of fresh food material. Therefore, the amount of moisture content in *V. doniana* and *S. comorensis* fruits can help to add amount of water in a body for a healthy skin, digestion and good flow of blood in the body.

### Variation of ash content in the fruits of *V. doniana and S. comorensis*

The total ash contents observed in *S. comorensis* are similar from that reported by^23^ on the edible wild fruits from Malawi that had percent of ash content in a range from 3 to 5%. *S. comorensis* from coastal forests had higher percent of ash content than that reported by^24^ in other *Saba* species for example *Saba senegalensis* have very low ash content (2.80%). Amarteifio and Mosase^25^ reported the ash content in *S. birrea* (4.9%) and *V. infausta* (3.9%) that correlated with the value observed in this study in *S. comorensis*. The ash content in *S. comorensis* were similar to those reported by^26^ that ranged from 3 to 7.8% in *A. digitata* pulp and kernel.

Ochieng et al.^20^ reported an amount of total ash in *V. doniana* (3.41 ± 0.09%) which is higher than ash content in *V. doniana* fruits samples reported in this study. These results are not similar to those reported on *V. doniana* by^23,27^ which were 4.8% and 5.27% respectively. The ash content in *V. doniana* were also similar to those reported by^26^ that ranged from 3 to 7.8% in *A. digitata* pulp and kernel. Amarteifio and Mosase^25^ reported the ash content in *S. birrea* (4.9%) and *V. infausta* (3.9%) that correlated with the ash content observed in *V. doniana* in this study.

Ash content helps to determine the amount and type of minerals in food sample, as well as retard the growth of microorganism^28^. Ash content determination is a part of proximate analysis for nutritional evaluation and for preparation of a food sample for a specific elemental analysis^28^. Minerals are involved in the formation of bones and teeth, essential constituency of body fluids and tissues. This range of ash content shows that *S. comorensis* and *V. doniana* can be alternative sources of essential mineral nutrients.

### Variation of protein content in the fruits of *V. doniana and S. comorensis*

Most wild fruits have a good value of protein content^24^. *V. doniana* results obtained are comparable to that reported by^29^ in *Maerua pseudopetalosa* (19.26% to 22.06%) and similar from that reported in *Sterculia Africana* oil (24.90 ± 0.63%) by^30^. *V. doniana* which had 7% of crude protein which is higher compared to that reported by^23^ with 2.6%. These values indicate that fruits may not be an excellent source of protein. However, *S. comorensis* from Tanzania have relatively lower protein content than some fruits but also higher than *S. senegalensis* (0.53%) reported by^24^. In this study, the protein content of *S. comorensis* is comparable to that of *Mangifera indica* (4.01 mg/100 g) reported by^22^ but higher than *Passiflora edulis* (0.90 mg/100 g) reported by^12^ and *Citrus sinensis* (0.8 mg/100 g) reported by^13^. Kwua and Orahb^31^ reported *Dennettia tripetala* fruits to have protein content of 15.3 g/100 g which is higher than that of *S. comorensis*. However^32^, reported a protein content of indigenous fruits of South Africa as 8.2 g/100 g. Higher amount of protein in *S. comorensis* and *V. doniana* showed significant difference at *P* < 0.05. These differences in protein content can be ascribed to environmental factors and more likely by extraction procedures where during separation of the pulp and seeds, some small quantity of substituents from seed can still be mixed up. Seeds from different studies reported to have high amount of protein than the fruit pulp. It can therefore be said that while *S. comorensis* and *V. doniana* are not very good sources of protein, it is still worthy eating these wild fruits in order to get their other nutritional benefits while complementing proteins from other food sources.

### Variation of crude fat content in the fruits of *V. doniana and S. comorensis*

The range of fat content in most edible fruits is reportedly less than 0.5 g/100 g^24^. Fats help to maintain body temperature and make up all body cells. Apart from other sources of energy such as carbohydrate, fats provide high levels of energy and they are also reservoirs of fat-soluble vitamins. The crude fat content of *V. doniana* and *S. comorensis* are presented in Table 1 and Table 2. This range of results presented in Table 1 and Table 2 is very similar to those reported by^18^ in *Cucumis melo*, *Psidium guajava*, *Carica papaya*, *Carissa carandas* which had 0.0084%, 0.023%, 0.02%, and 1.27% respectively*.* Other similar results are reported by^33^ in *Baccaurea sapida* (0.73%), *Morus alba* (0.21%) and *Terminalia chebula* (3.90%). *S. comorensis* had the lowest content below the detection limit (0.00 ± 0.01%) and (0.00 ± 0.00%) from Tanga and Pwani. The results are very similar to those reported in *Passiflora edulis* which had 0.00% by^12^.

*Vitex doniana* from Tanga had higher value of fat content (1.9 ± 0.10%) than those reported by^23^ in the same fruits that had 0.7%. Emmanuel et al.^30^ reported *V. mambossae, A. digitata, Opilia amentacea* to have high values of fat contents 2.97 ± 0.29%, 3.88 ± 0.13% and 2.45 ± 0.40% respectively, which are higher than those in *V. doniana* from Pwani and Tanga having the value of 2.4 ± 0.00% and 1.9 ± 0.10% respectively. It can be noted that *S. comorensis* have low amounts of fats. Low fat foods are considered healthy because high consumption of fats is linked to health problems such as obesity, cardiovascular diseases, higher blood pressure, stroke, breathing problem and higher cholesterol.

### Variation of total carbohydrate content in the fruits of *V. doniana and 5 S. comorensis*

The total carbohydrate in *S. comorensis* and *V. doniana* were higher than those of domesticated fruits such as *Mangifera indica* (0.0 mg/100 g) reported by^22^ and *Citrus sinensis* (6.0 mg/100 g) reported by^13^. The mean value of 7% and 21% of *V. doniana* from Tanga and Pwani regions is however lower than 29.57 ± 0.07% reported by^20^ for the same fruits collected from Kenya. However, the amount of carbohydrate in this study from *S. comorensis* were lower than those of *S. senegalensis* (74.23%) which is of the same genus^24^. *S. comorensis* and *V. doniana* from Pwani had higher total carbohydrate of 23.81 ± 0.38% and 23.98 ± 0.20% respectively than those found from Tanga. The amount of cabohydtrate in *S. comorensis* and *V. doniana* from Pwani region were higher than those reported by^18^ for the exotic fruits *Psidium guajava* (15.52 g) and *Carica papaya* (10.93 g).

In this study, total carbohydrate content of *V. doniana* fruits ranged from 23.78 to 24.18% for fruit samples from Pwani and 8.32–9.12% for fruit samples from Tanga. The differences may be attributed to different soils or other environmental and climatic factor. Different environmental stresses or favorable conditions may affect the metabolic efficiency of plants in those areas and hence perform differently when it comes to the metabolites they produce^34^. The amount of carbohydrate determined indicated that wild fruits can be an important source of dietary energy. The amount of carbohydrate in wild fruits contribute to higher calorific value compared to the exotic fruits. Findings from this study recommend the increase consumption of *S. comorensis* and *V. doniana* which may serve as valuable source of energy to human body.

## Conclusion

Based on the results from this study wild fruits especially *S. comorensis* and *V. doniana* very nutritious having vital nutrients. However, there is no single fruit that can provide all adequate nutrients required by human being yet these wild fruits have many essential comprising nutrients such as carbohydrate, protein, crude fats, ash and moisture content. They are therefore, very potential in contributing to human health by complementing to some of the nutritional inadequacies of some of the exotic fruits.

## Data Availability

All data are contained within this article.
